# Iowa

**Published:** 1898-05

**Authors:** 


### IOWA VETERINARY SOCIETY.

ANENT the annual meeting at Des Moines, Iowa, in January, between thirty and forty members responded to roll-call. The social and political standing of the profession found much expression in the President's address, which was fruitful in provoking earnest discussion.

The Association added twelve new members to its strength.

State Fair delegates mingled with the Association members in their evening session.

Dr. A. T. Peters, of Lincoln, Nebraska, addressed the Association on "Immunity," and made a strong plea for a larger attendance at Omaha.

State Veterinarian Gibson aroused much interest in State work through his report.

Many of the members attended the banquet of the State Fair Association.

Actinomycosis and immunity were strong in discussion on the second day.

The veterinary journals came in for a roasting because they would not furnish reprints without cost in exchange for a full report of the meeting.

Members are not to copyright their papers, but the latter and all reports are to become the property of the Association.

Dr. Parslow's paper on "Reflex Paralysis" proved to be an excellent presentation of the subject, and brought forth a profitable discussion.

Dairy inspection found many interested listeners, and its importance was fully presented by Dr. J. W. Griffith.

Senator Emmett presented the subject of proposed legislation to prevent the admission of dairy cattle into Iowa afflicted with tuberculosis. Also to better control the diseases of swine.

No effort will be made this session to secure legislation regulating the practice of veterinary science.

Governor Shaw's inauguration attracted the members on the afternoon of the second day.

The weight of opinion seemed in favor of removal of the clitoris in place of ovariotomy for viciousness in mares, thus preventing their loss for breeding purposes.

Some of the members came in for a round scoring in not practising what they preach relative to the personal use only of milk from tested cows free from tuberculosis.

The next meeting will be in connection with the Nebraska State Association at the convening of the U. S. V. M. A. in September.

